# A genome-wide scan to identify signatures of selection in two Iranian indigenous chicken ecotypes

**DOI:** 10.1186/s12711-021-00664-9

**Published:** 2021-09-09

**Authors:** Elaheh Rostamzadeh Mahdabi, Ali Esmailizadeh, Ahmad Ayatollahi Mehrgardi, Masood Asadi Fozi

**Affiliations:** Department of Animal Science, Faculty of Agriculture, Shahid Bahonar University of Kerman, 22 Bahman Blvd, Kerman, Iran

## Abstract

**Background:**

Various regions of the chicken genome have been under natural and artificial selection for thousands of years. The substantial diversity that exits among chickens from different geographic regions provides an excellent opportunity to investigate the genomic regions under selection which, in turn, will increase our knowledge about the mechanisms that underlie chicken diversity and adaptation. Several statistics have been developed to detect genomic regions that are under selection. In this study, we applied approaches based on differences in allele or haplotype frequencies (*F*_ST_ and hapFLK, respectively) between populations, differences in long stretches of consecutive homozygous sequences (ROH), and differences in allele frequencies within populations (composite likelihood ratio (CLR)) to identify inter- and intra-populations traces of selection in two Iranian indigenous chicken ecotypes, the Lari fighting chicken and the Khazak or creeper (short-leg) chicken.

**Results:**

Using whole-genome resequencing data of 32 individuals from the two chicken ecotypes, approximately 11.9 million single nucleotide polymorphisms (SNPs) were detected and used in genomic analyses after quality processing. Examination of the distribution of ROH in the two populations indicated short to long ROH, ranging from 0.3 to 5.4 Mb. We found 90 genes that were detected by at least two of the four applied methods. Gene annotation of the detected putative regions under selection revealed candidate genes associated with growth (*DCN*, *MEOX2* and *CACNB1*), reproduction (*ESR1* and *CALCR*), disease resistance (*S1PR1*, *ALPK1* and *MHC-B*), behavior pattern (*AGMO*, *GNAO1* and *PSEN1*), and morphological traits (*IHH* and *NHEJ1*).

**Conclusions:**

Our findings show that these two phenotypically different indigenous chicken populations have been under selection for reproduction, immune, behavioral, and morphology traits. The results illustrate that selection can play an important role in shaping signatures of differentiation across the genomic landscape of two chicken populations.

**Supplementary Information:**

The online version contains supplementary material available at 10.1186/s12711-021-00664-9.

## Background

Chickens are raised for different purposes, including meat and egg production, as well as for entertainment, in diverse geographical areas. Several studies have reported the multiple times when and multiple places where the domestication of chicken from the Jungle fowl species occurred. However, most of these studies suggested that domestic chicken originated from the Red Jungle fowl (*Gallus gallus*) in the south and Southeast Asia [1–4]. Selection pressures affect genome structure over time and leave signatures in specific regions of the genome, such as increased allele frequencies, extensive linkage disequilibrium, homozygous genotypes, and decreased local diversity [5–7]. In addition, forces such as genetic hitchhiking and background selection affect the genomic regions that are near the sites that are under selection [8]. In order to identify such signatures of selection, different statistical methods have been developed, which are generally categorized into two groups based on whether signatures of selection are investigated within populations (intra-population) or between populations (inter-population). Among the methods for within-population studies of signatures of selection, some are based on runs of homozygosity (ROH) [9], pooled heterozygosity (Hp) [10], integrated haplotype scores (iHS) [11], and composite likelihood ratio (CLR) [12]. Methods for between-population studies are based on the fixation index (*F*_ST_) [13], cross-population extended haplotype homozygosity (XP-EHH) [7, 14] and hapFLK [15]. Since each of these statistical tests identifies specific patterns of selection in the genome, the joint use of different statistical tests has been suggested to increase the power of detection of signatures of selection along the whole genome [16, 17].

To decipher the genetic mechanisms that are involved in domestication and in the phenotypic differentiation of individuals belonging to the same species, detection of signatures of selection using different statistics has been performed in various species, including cattle [18, 19], sheep [20, 21], goats [22, 23], and chickens [24–26]. Most of the studies that explored selective footprints in chickens have used the single-site differentiation statistic commonly known as the fixation index, *F*_ST_, and have led to the identification of a range of candidates genes that contribute to adaptation to hot climates, reproduction [27], immune response, tolerance to harsh local environments [25], fat deposition, growth, skeletal development, and energy metabolism [28]. However, the *F*_ST_ method does not account for the hierarchical structure of populations and assumes that the subpopulations have derived independently from the same ancestral population [15]. To address this deficiency, the HapFLK method, an extension of the FLK statistic based on linkage disequilibrium (LD) [29], was introduced by Fariello et al. [15]. The HapFLK statistic computes signatures of selection by integrating both the hierarchical structure of populations and information on haplotype frequencies, which enhances the power of detection of signatures of selection. Application of the hapFLK approach has led to the detection of signatures of selection related to production and growth traits and to adaptation to extreme environments in non-commercial and commercial chickens [27, 30].

ROH are contiguous lengths of homozygous segments of the genome where the two identical haplotypes have likely been inherited from a common ancestor. Fleming et al. [25] employed an approach based on assessing the consensus ROH sequences between two indigenous chicken populations and revealed that different environments, as a driver of selective pressure, may play a role in the genomic divergence of populations. Another analysis of consensus overlapping ROH (cROH) identified genes that are involved in the immune system and homeostasis maintenance in a paternal broiler line [31]. ROH and *F*_ST_ mapping in African chicken breeds and indigenous ecotypes detected signatures of selection related to adaptation to heat, immunity, calcium ion binding, behavior, etc. [32].

Indigenous chickens are notable for their capacity to adapt to their respective climates and for their disease resistance and it is essential to preserve them as valuable genetic resources. Since Iranian indigenous chickens have never been subjected to selection programs for production traits, mapping the genes and quantitative trait loci (QTL) that are associated with such traits would be useful for future breeding programs. However, there is limited knowledge about the genomic regions and genes involved in economic traits of interest and in adaptation to harsh environment conditions [33].

There is a worldwide diversity of domesticated chickens that differ in morphology, physiology, and behavior [10]. Lari and Khazak are two indigenous ecotypes that are generally maintained in rural areas of Iran. Lari is known as a cockfighting bird and is generally distributed in different regions of Iran and has been selected for fighting characteristics by the locals. In the current study, the Lari samples were collected from the Fars province, situated in the Southern part of Iran, where the Lari ecotype is adapted to a hot and semi-dry climate. It is famous for some morphological and behavioral characteristics such as its large body size, pugnacity, high stamina, and aggressive behavior. Its body shape is unique and different from all other indigenous Iranian ecotypes. The standing posture, long neck (average neck length of 17 cm), and long legs (on average 16 cm) make it look larger. Its mature body weight is about 3 to 4 kg. Lari chicken produce only 65 to 70 eggs per year. The Khazak ecotype lives in southeastern Iran (Zabol Province), where the climate is hot and dry. It is mostly raised for egg production. Its distinctive feature is its short legs (on average 4 cm), such that it is known as a creeper chicken in the local language. Its mature body weight is around 1.2 to 1.5 kg. The Khazak ecotype produces 120 to 130 eggs per year [34, 35]. Comparison of the body weight and egg production between these two ecotypes indicates that Lari is more like a broiler while Khazak is more like a layer. The aim of this study was to explore the genomic differences between these two indigenous ecotypes by detecting the regions or sites under natural or artificial selection using resequencing data and four statistical approaches, i.e. *F*_ST_, ROH, hapFLK, and CLR.

## Methods

### Sampling and genome sequencing

Thirty-two chickens were sampled from the south and southeastern regions of Iran (15 Lari and 17 Khazak). Each ecotype was sampled in multiple village flocks in order to minimize family structure or high levels of co-ancestry between individuals. Extraction of genomic DNA from blood collected in EDTA-coated tubes was carried out using the standard phenol–chloroform protocol [36]. Quantity and quality of each DNA sample were evaluated using an ultraviolet spectrophotometer and agarose gel electrophoresis, respectively. Ten µg of genomic DNA were used to construct libraries with a 350-bp insert size according to the Illumina library preparation pipeline and sequenced on the Illumina Hiseq 2000 platform to generate 125-bp paired-end reads (Illumina Inc., USA). Before analysis, the raw sequences were checked by the FastQC tool [37] and low-quality reads and adaptors were trimmed out using the Trimmomatic software [38], with default parameters.

### Sequence alignment and single nucleotide polymorphism calling

Paired-end reads were aligned to the chicken reference genome (Galgal6.101) using the mem algorithm in the Burrows-Wheeler Aligner (BWA) software [39], the resulting files were sorted using SAMtools [40], and PCR duplicates that were created during the preparation of genomic libraries were marked and removed by Picard tools (http://broadinstitute.github.io/picard/). In order to improve the accuracy of downstream processing steps, local alignment of the sequences around InDels was performed by RealignerTargetCreator and IndelRealigner in the GATK software [41]. To adjust and improve the base quality scores, we applied BaseRecalibrator and PrintReads, and finally, single nucleotide polymorphism (SNP) calling and hard-filtering were performed using "UnifiedGenotyper" and "VariantFiltration" arguments, respectively, in GATK. We removed the SNPs that were located on sex chromosomes and those that were not assigned to a specific linkage group. The multisampling VCF file was converted to Plink format and the Plink 1.9 software [42] was used to remove individuals with 10% missing genotypes (zero), and SNPs with a Hardy–Weinberg equilibrium test P-value below the 10e−6 threshold (16,143 SNPs), a genotyping rate lower than 90% (1,020,648) and a minor allele frequency less than 5% (4,377,724 SNPs).

### Population structure

To investigate the genetic structure and differentiation of the target populations, a phylogeny tree was created by the maximum likelihood method using the PHYLIP package in SNPhylo [43]. A principal component analysis (PCA) was implemented on the filtered SNP data using R functions in the SNPRelate package [44].

### Intra-population signatures of selection

#### Composite likelihood ratio (CLR)

The CLR test detects differences in allele frequencies along a chromosome between a neutral and a selective sweep model, which is able to detect variants that are close to fixation [45]. We performed a genome-wide scan using Sweepfinder2, which implements a likelihood-based method [46] to calculate the CLR statistic for each site with a 20-kb grid size across the genome in each population. Putative regions under selection were obtained by dividing the genome into windows of 200 kb. Following a previous approach [47], the maximum CLR was applied as the test statistic and the top 1% regions of the empirical distribution were deemed significant selective sweeps.

#### Run of homozygosity (ROH)

Putative genomic regions under selection within each population were detected by the “Runs of homozygosity” function of the Plink software. The parameters were set according to Ceballos et al. [48], such that a minimum number of 50 SNPs was set to determine ROH. To prevent the underestimation of long ROH regions and also considering genotyping errors, three heterozygous positions and five missing SNPs were allowed per window. The length of the sliding window was set to 300 kb. A minimum density of 1 SNP per 50 kb was required to consider a ROH and the proportion of homozygous overlapping windows was set to 0.05. The parameter homozyg-group in the Plink software was used to identify overlapping ROH (pools) based on the threshold of an allelic match with 95% identity. When a homozygous consensus sequence was detected in more than five individuals of each ecotype within the same region of the genome in a pool, it was considered as a putative region under selection and gene annotation and enrichment analysis were performed for the genes located in these regions.

### ROH distribution and inbreeding coefficients

The frequencies and length of ROH across the genome can differ between individuals. For instance, a longer ROH implies recent inbreeding, while a shorter ROH indicates more ancient common ancestors in the pedigree [48, 49]. We calculated ROH and inbreeding coefficients for different classes of ROH length: short (0.3 to 1.0 Mb), medium (1.0 to 1.5 Mb), and long (> 1.5 Mb) per animal using the runs of homozygosity parameter in the Plink software and the method defined by McQuillan et al. [9]:

$$F_{ROH} = \frac{{L_{ROH} }}{{L_{auto} }},$$

where ${L}_{ROH}$ is the sum of the genomic lengths covered by ROH per animal and ${L}_{auto}$ is the autosomal genome length covered by SNPs.

### Inter-population genetic differentiation

#### Wright’s fixation index (F_ST_)

We compared two indigenous chicken populations that were phenotypically different. The *F*_ST_ statistic, which is a method based on the differentiation in allele frequencies between populations, was used to identify genomic footprints of selection between the two populations. This approach has been applied in numerous studies to investigate the underlying genomic mechanism of variation between populations [26, 50]. To calculate the per-site *F*_ST_ statistic between the two populations, we used the VCFtools software [51], based on Weir and Cockerham's *F*_ST_ estimator [52]. Pairwise *F*_ST_ values were estimated in 50-kb sliding windows along each chromosome, with a step size of 25 kb. The degree of genetic divergence and differentiation between populations (*F*_ST_) ranges from 0 to 1, where values close to 0 refer to a low genetic differentiation, i.e. the two populations have no genetic differentiation and share many fixed loci and values close to 1 refer to a stabilized difference between populations [53]. In this study, negative *F*_ST_ values were set equal to zero because negative values do not have a biological interpretation [54]. We considered the top 1% of the empirical distribution of mean *F*_ST_ (m*F*_ST_) values as divergent selection footprints.

#### hapFLK test

The hapFLK statistic which is a haplotype-based approach, was calculated according to the methodology described by Fariello et al. [15] using the hapFLK software that is available at https://forge-dga.jouy.inra.fr/projects/hapflk. We used 10 clusters (-K 10) for the fastPHASE cross-validation procedure to obtain haplotype diversity [55]. The hapFLK statistic was assessed as the average across 15 expectation–maximization runs (-nfit = 15) to fit the linkage disequilibrium model.

Since hapFLK values for each SNP along the genome approximately follow a normal distribution, to robustly evaluate the normal distribution parameters, hapFLK values were standardized to evaluate the p-value for each SNP using the formula:

$$hapFLK_{adj} = \frac{{hapFLK - Mean\left( {hapFLK} \right)}}{{SD\left( {hapFLK} \right)}},$$

as implemented in the rlm function of the R MASS package, where $Mean(hapFLK)$ and $SD(hapFLK)$ refer to the mean and the standard deviation of hapFLK values across the genome, respectively. To limit the number of false positives, the false discovery rate (FDR) was estimated using the R Bioconductor qvalue package [56]. SNPs that reached an FDR threshold of 1% were considered significant (− log10(P-value) = 5.19).

### Gene set enrichment analysis

To control and avoid the detection of likely false positives by different methods, we focused on signatures of selection that were identified by at least two of the four approaches and on those that were found in peak points in the hapFLK analysis as putative signatures of selection. The genes located in these regions were determined by the Variant Effect Predictor tool (release 101) [57] and functional annotation of the candidate genes, molecular functions, and biological processes was carried out using the Database for Annotation Visualization and Integrated Discovery (DAVID) [58]. Applying the Fisher exact statistics, a P-value < 0.05 was considered for the statistical significance of GO term enrichments.

## Overlap of signatures of selection with reported QTL

In order to examine the overlap of the putative genomic regions under selection with previously known QTL, we downloaded the QTL data from the chicken QTL database (http://www.animalgenome.org/cgi-bin/QTLdb/GG/index). Then, we used the regioneR [59] package and the given region coordinates (chromosome, start and end) to identify reported QTL that were located in the putative regions under selection. However, because of the limited resolution of the QTL localizations, many overlaps are expected to occur by chance. Thus, we used a permutation test (n = 1000) to identify statistically significant associations between putative regions and the QTL regions that were repeatedly sampled by the permutation test.

## Results

### Sequence alignment and SNP calling

After quality control and trimming, ~ 64 million reads per individual remained. A high percentage (98.0%) of the short reads were properly mapped to the chicken reference genome (galgal6). The average sequencing depth for the 32 individuals was 7.1 (6.9 for Khazak and 7.3 for Lari). In total, 17,287,526 SNPs were detected from the whole-genome data in the initial SNP calling. After quality control, 11,873,011 SNPs across all individuals were retained. Of those, about 6.5 million SNPs were shared between the two ecotypes, and 2.9 and 2.8 million SNPs were specific to the Lari and Khazak ecotypes, respectively.

### Population structure analysis

**Fig. 1:**
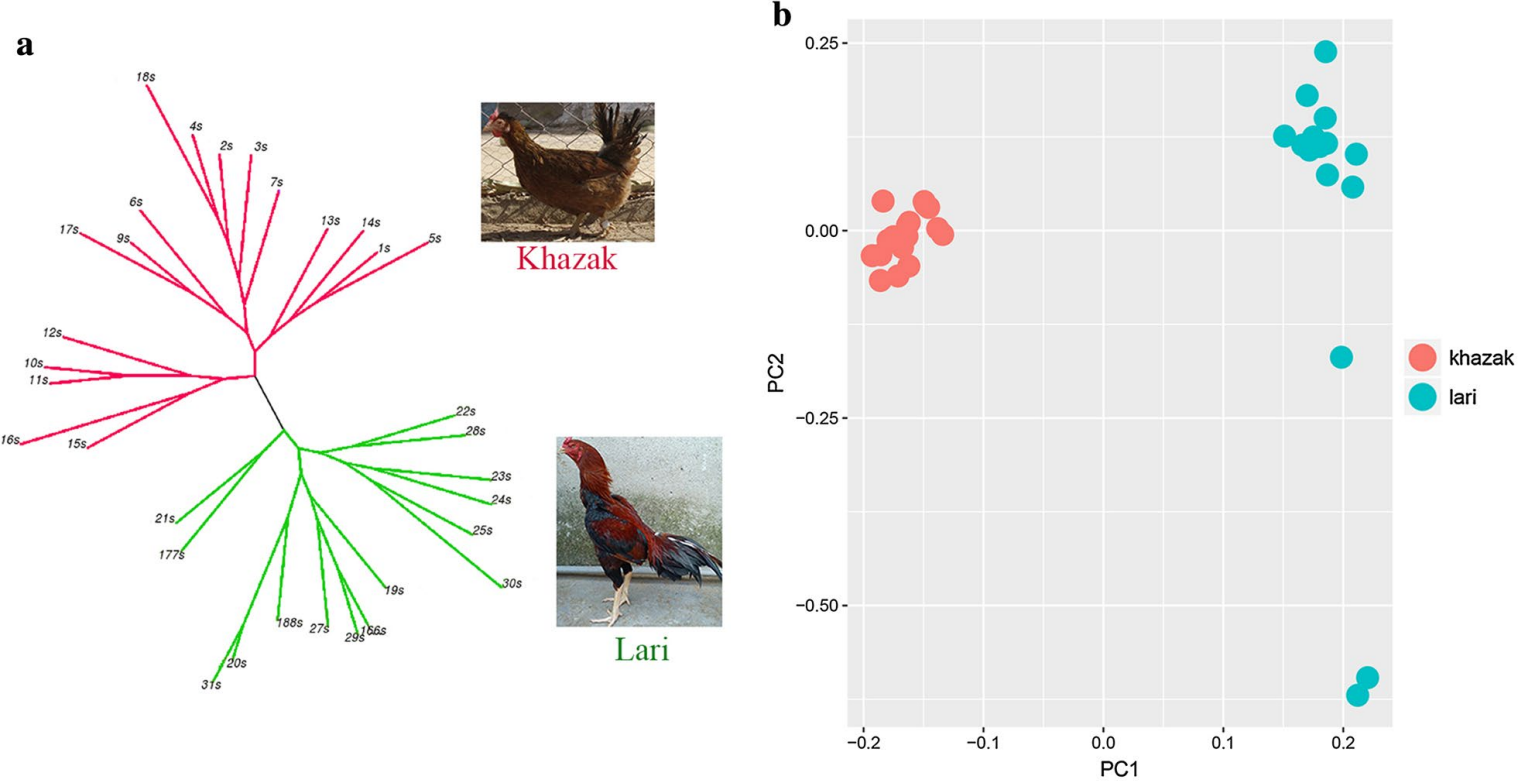
**a** Maximum likelihood tree based on the genotype data, in which the clusters of the Khazak and Lari ecotypes are separated by red and green lines, respectively, and **b** principal component analysis of the genotype data from the two populations

Using the 11,873,011 SNPs, a maximum likelihood phylogenic tree was constructed and the evolutionary divergence of populations was visualized. Although there were sub-clusters in each population, the two populations segregated into two distinct clusters (Fig. 1a). Population structure, which was evaluated by principle component analysis based on genetic relationships between individuals, classified the birds into two distinct populations, i.e. Lari and Khazak. The PCA results (Fig. 1b) validated those obtained by the phylogenic tree.

### Runs of homozygosity and inbreeding

**Fig. 2:**
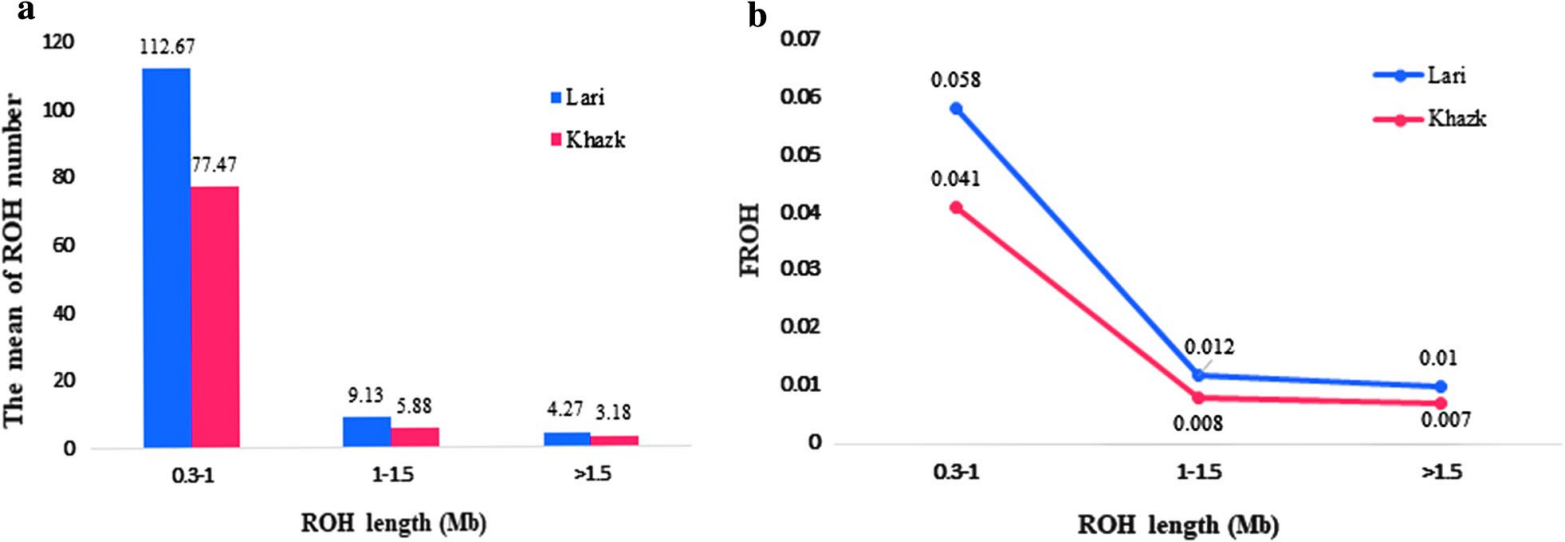
a The mean of runs of homozygosity number and **b** distribution of inbreeding coefficients (*F*_ROH_) values in the short, medium, and long categories

In the ROH analysis, 3362 ROH segments were found, with an average of 105.1 ROH per animal (see Additional file 1: Table S1). The average genomic length covered by ROH per animal was 50.416 Mb and 74.233 Mb for the Khazak and Lari ecotypes, respectively. Consecutive lengths of homozygous genotypes were observed on all chromosomes, except chromosomes 16, 30, 31, 32, and 33. About 70% of the ROH segments were located on macro-chromosomes, while ROH that were more than 5 Mb long were on chromosome 4 (71,896,991–77,295,695 bp and 61,312,016–66,772,831 bp). The average ROH coverage was generally higher for short ROH segments (i.e. shorter than 1.0 Mb), with a mean coverage of 45.717 Mb per animal, than for long ROH segments (i.e. longer than 1.0 Mb), with a mean coverage of 16.730 Mb per animal (Fig. 2). The number of long ROH (> 1.0 Mb) per animal was smaller than the number of short ROH (< 1.0 Mb) for both ecotypes (Fig. 2a). The average number of ROH segments per individual was larger for the Lari ecotype (126.0 ROH per individual) than for the Khazak ecotype (89.5 ROH). Consequently, the Lari showed a higher inbreeding coefficient (*F*_ROH_) than the Khazak ecotype (Fig. 2b). For both ecotypes, the highest genomic inbreeding coefficient was found for the 0.3–1 Mb ROH category (Fig. 2b).

The values of genome-wide inbreeding coefficients differed between the two ecotypes, with the highest, mean and lowest individual *F*_ROH_ values equal to 0.28, 0.078, and 0.049, respectively, for the Lari ecotype, and 0.125, 0.056, and 0.015 for the Khazak ecotype.

### Intra-population signatures of selection

#### CLR statistic

**Fig. 3:**
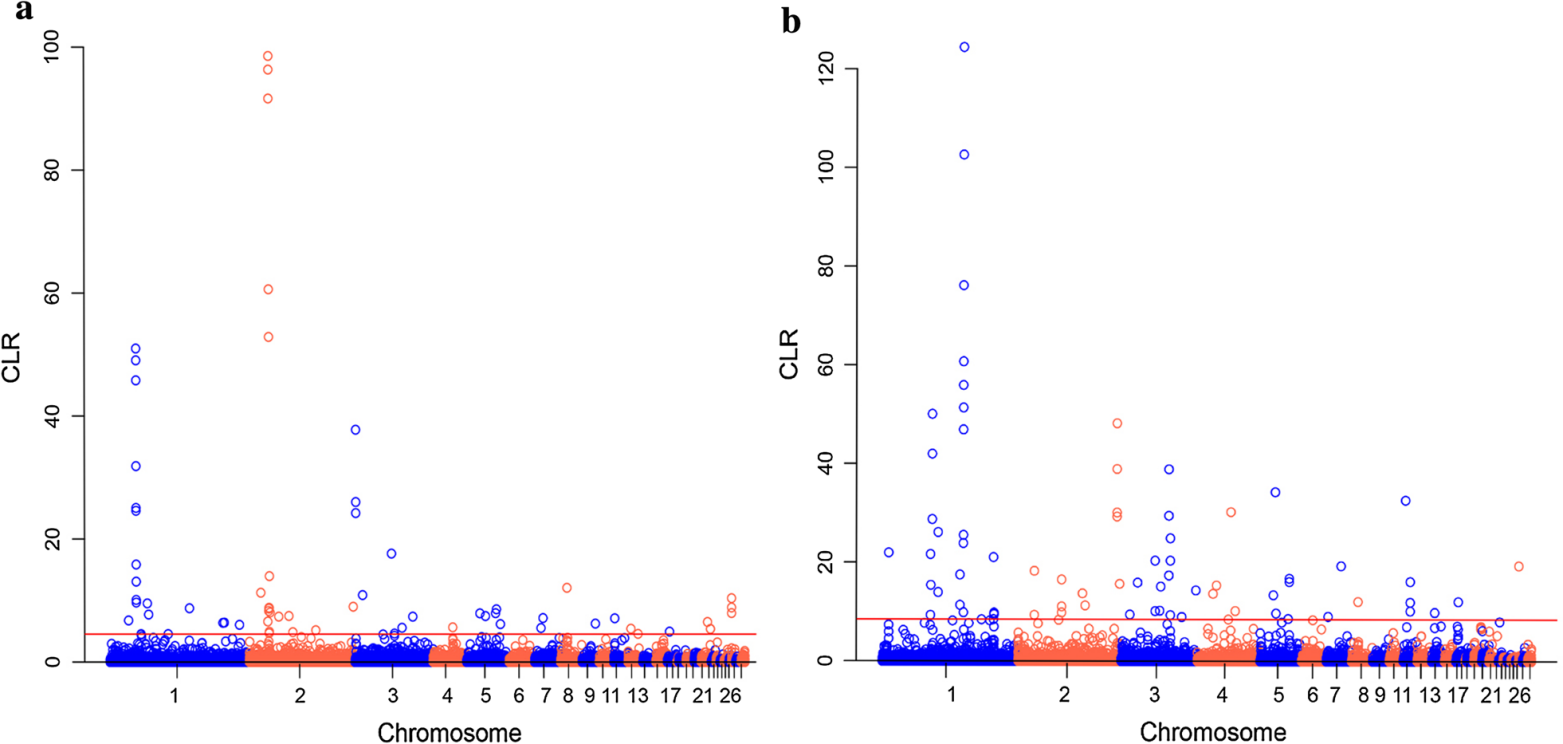
Distribution of composite likelihood ratio values along the autosomal chromosomes of the Lari (**a**) and Khazak (**b**) chickens. The red line corresponds to the top 1% of the empirical distribution of CLR values

We computed the CLR test to detect recent selective sweeps in the Lari and Khazak ecotypes. The distribution of CLR values is illustrated in Fig. 3 and revealed significant genomic positions in the top 1% CLR scores detected in the Lari (CLR > 4.54) and Khazak (CLR > 8.47) ecotypes. The most significant CLR value in the Lari ecotype (98.56) was observed on chromosome 2, in a region between 27.6 and 27.8 Mb, which harbors the *DGKB* (*diacylglycerol kinase beta*) gene (Fig. 3a), while the most significant CLR value in the Khazak ecotype (124.43) was observed on chromosome 1 between 117.6 and 117.8 Mb (Fig. 3b).

We obtained 342 candidate genes for both ecotypes, of which 225 were protein coding genes (see Additional file 2: Table S2). Only seven genes were shared between the two ecotypes. Twenty six and eight genes were shared with the annotated genes in the *F*_ST_ and ROH analyses, respectively (Table 1) and (see Additional file 3: Table S3). Some of the candidate genes identified in the Khazak ecotype have functional relevance to reproduction (*ELF3*, *ESR1*, and *CALCR*), biosynthesis of fatty acid and abdominal fat deposition (*ELOVL2* and *MAOA*), and immune traits (*DOCK2*, *LCP2*, *PTPN2* and *IL1RAPL1*), while those identified in the Lari chickens are involved in regulation of energy homeostasis (*AGRP*), immune (*APBB1IP*), muscle development (*HDAC9*), wound healing (*MMP13*), metabolic regulation and reproduction (*TSHR*), and behavioral traits (*AGMO* and *PSEN1*). In the CLR test, enriched GO terms were revealed for the biological process, cellular components, and molecular functions categories. Functional terms included those involved in epithelial cell proliferation, Golgi organization, and cell differentiation (see Additional file 4: Table S4).

**Table 1:** Summary of the genes that were identified by at least two of the four methods used *F*_ST_, hapFLK, composite likelihood ratio and run of homozygosity

Chr	Gene ID	Gene symbol	Gene start (bp)	Gene end (bp)	Method
1	ENSGALG00000011274	*DCN*	44,050,178	44,202,174	*F*_ST_, ROH-L
1	ENSGALG00000012559	*LARGE1*	52,678,868	52,954,337	*F*_ST_, CLR-L
1	ENSGALG00000016288	*IL1RAPL1*	117,527,572	11,8143,733	CLR-K, ROH-L-K
2	ENSGALG00000008591	*CACNB2*	19,119,896	19,343,839	hapFLK, CLR-L
2	ENSGALG00000009509	*CALCR*	23,060,807	23,197,789	*F*_ST_, CLR-K
2	ENSGALG00000010792	*AGMO*	27,838,091	28,021,352	*F*_ST_, CLR-L, ROH-L
2	ENSGALG00000010794	*MEOX2*	28,039,372	28,092,164	*F*_ST_, CLR-L, ROH-L
2	ENSGALG00000010854	*HDAC9*	29,076,224	29,386,193	*F*_ST_, CLR-L
2	ENSGALG00000013817	*SPIRE1*	97,073,307	97,195,740	*F*_ST_, CLR-K
3	ENSGALG00000012973	*ESR1*	49,053,965	49,241,576	CLR-K, ROH-K
4	ENSGALG00000012074	*ALPK1*	57,133,859	57,167,350	*F*_ST_, ROH-L
5	ENSGALG00000009320	*PSEN1*	26,593,233	26,614,911	*F*_ST_, CLR-L
8	ENSGALG00000005208	*S1PR1*	12,067,120	12,071,019	*F*_ST_, ROH-L
20	ENSGALG00000008010	*PTPN1*	13,537,599	13,576,469	*F*_ST_, CLR-K

Chr: chromosome number

#### Consensus ROH

In each ecotype, ~ 87% of the consensus ROH were shared among two to five animals, while 176 consensus ROH pools that were common to at least five individuals (more than 30% of the individuals in each ecotype) were identified (110 in the Lari and 65 in the Khazak population) (see Additional file 5: Table S5). The consensus ROH were located on chromosomes 1, 2, 3, 4, 5, 7, 8, 9, 11, 15, 17, 19 and 20 in the Khazak and on chromosomes 1, 2, 3, 4, 5, 6, 7, 8, 11, 12, 13, 14, 15 in the Lari chickens.

Annotation analysis of consensus ROH revealed 282 protein coding genes (see Additional file 6: Table S6), of which 30 were shared between the two ecotypes (Table 1) and (see Additional file 3: Table S3). One consensus ROH on chromosome 2 (~ 347 kb: 27,726,057–28,073,095 bp), was shared by 11 of the 15 individuals of the Lari ecotype and included the *AGMO* (*alkylglycerol monooxygenase*) gene. For the Khazak population, one consensus ROH was identified on chromosome 8 (~ 84 kb: 654,688–738,425 bp) that was common to 16 individuals and included long non-coding RNA transcripts.

Seven biological process GO terms were significantly enriched among the genes located in consensus ROH regions (3 in Lari and 4 in Khazak), of which some contained the terms axonogenesis (GO: 0007409), visual perception (GO: 0007601), blood vessel maturation (GO: 0001955), and response to hypoxia (GO: 0001666) (see Additional file 4: Table S4). Several other genes that were annotated in consensus ROH were involved in muscle tissue development and regeneration, osteoblast differentiation, breast muscle development, cardiac muscle tissue growth involved in heart morphogenesis, and cardiac muscle contraction. We also observed many genes in the consensus ROH that have a role in the innate and adaptive immune system, B cell differentiation, T cell activation involved in immune response, interleukin-17 production, toll-like receptor 3 signaling pathway, and interleukin-4 production regulation, regulation of the interleukin-6 biosynthetic process, and inflammatory response. Some of the genes in the consensus ROH analysis were linked to behavioral traits, including feeding, social, and aggressive behavior, and productive traits such as spermatogenesis, ovarian follicle development, and spermatogenesis (see Additional file 4: Table S4).

### Inter-population genetic differentiation

#### F_ST_ statistic

**Fig. 4:**
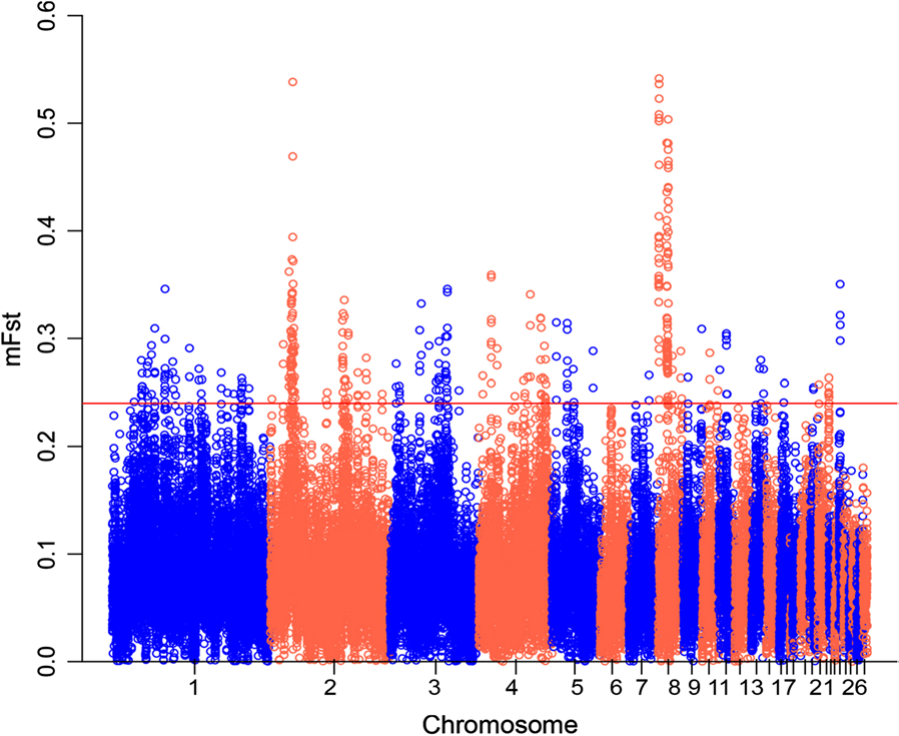
Distribution of *F*_ST_ values, showing signatures of differentiation between the Lari and Khazak chickens. The dashed black line corresponds to the top 1% of the empirical distribution of mean *F*_ST_ values

Using 25-kb sliding windows, 374 regions with an m*F*_ST_ higher than 0.24 were in the top 1% of the empirical distribution. These regions were located across most of the chicken chromosomes. The highest *F*_ST_ values belonged to an intergenic region on chromosome 8 (875,001–925,000 bp) (m*F*_ST_ = 0.541) and a protein-coding region on chromosome 2 (27,825,001–27,875,000 bp) that spans the *AGMO* gene (*F*_ST_ = 0.538) (Fig. 4). Enrichment analysis of the genes covered significant GO terms related to biological functions, molecular processes, and cellular components related to double-strand break repair, developmental process, vocal learning, transcriptional activator activity, RNA polymerase II core promoter proximal region sequence-specific binding, nucleoplasm and Golgi apparatus (see Additional file 4: Table S4).

In total, 184 protein-coding genes were detected in the putative regions under selection (see Additional file 7: Table S7), among which 54 and 26 genes were common to the annotated genes in the ROH and CLR analyses, respectively (Table 1) and (see Additional file 3: Table S3). The results derived from the gene annotation analysis showed that multiple genes were involved in the immune system, such as *TRIM13* (*tripartite motif-containing 13*), *ALPK1* (*alpha kinase 1*), *S1PR1* (*sphingosine-1-phosphate receptor 1*), and *ITK (IL2-inducible T-cell kinase*), which mapped to chromosomes 1, 4, 8, and 13, respectively. Furthermore, our results indicate that several genes in the detected regions are associated with growth and reproductive traits, such as *DCN* (*decorin*), *MEOX2* (*mesenchyme homeobox 2*), *HDAC9* (*histone deacetylase 9*), *SGCZ* (*sarcoglycan, zeta*), *LARGE* (*like-glycosyltransferase*), and *CITED4 (Cbp/p300-interacting transactivator, with Glu/Asp-rich carboxy-terminal domain, 4*) (see Additional file 7: Table S7).

#### hapFLK statistic

**Fig. 5:**
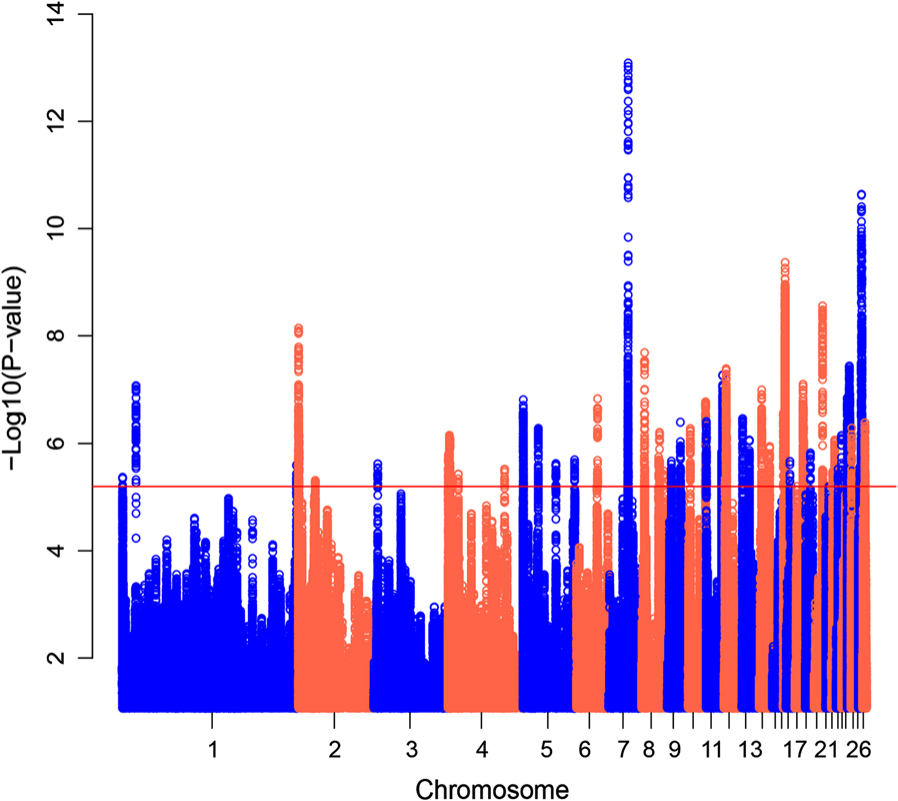
Distribution of hapFLK values along autosomal chromosomes of the Lari vs. the Khazak chickens. The red line shows the FDR threshold < 0.01

Results of the hapFLK test are shown in Fig. 5 and Table 2. A threshold of P-value < 6.3 × 10^–6^ was used to declare genomic regions that have been under selection. We observed signatures of selection on all the autosomes except 15, 21, and 24. The strongest signals were detected on chromosomes 7 (22.05–22.35 Mb), 27 (6.48–7.69 Mb), and 16 (2.40–2.75 Mb).

**Table 2:** Summary of the regions of putative signatures of selection detected by hapFLK analysis

Chr	Position (Mb)	Number of significant SNPs	Peak P-value	Peak Q-value	Number of genes
1	434,470–434,352	5	4.27 × 10^–6^	8.11 × 10^–3^	–
1	15,481,982–15,485,698	59	8.56 × 10^–8^	9.62 × 10^–4^	2
1	195,604,292–195,605,428	19	2.52 × 10^–6^	6.11 × 10^–3^	3
2	320,222–905,567	318	7.26 × 10^–9^	2.5 × 10^–4^	19
2	19,149,170–19,168,337	9	4.90 × 10^–6^	8.74 × 10^–3^	1
3	4,315,154–4,316,471	28	2.40 × 10^–6^	5.89 × 10^–3^	1
4	592,005–1,129,104	33	1.36 × 10^–6^	4.73 × 10^–3^	2
4	1,793,886–2,540,113	162	7.05 × 10^–7^	2.98 × 10^–3^	6
4	11,848,194–11,848,517	11	3.72 × 10^–6^	7.53 × 10^–3^	1
4	63,734,725–63,735,252	10	3.03 × 10^–6^	6.72 × 10^–3^	1
5	352,470–658,990	128	1.56 × 10^–7^	1/39 × 10^–3^	11
5	17,260,400–17,270,064	72	5.24 × 10^–7^	2.57 × 10^–3^	5
5	36,923,176–57,911,202	58	2.02 × 10^–6^	5.32 × 10^–3^	2
6	23,492,639–24,056,037	45	1.49 × 10^–7^	1.36 × 10^–3^	3
7	22,056,870–22,351,378	471	9.51 × 10^–14^	2.84 × 10^–7^	15
8	4,089,299–4,679,692	91	2.07 × 10^–8^	4.27 × 10^–4^	16
8	20,142,893–25,633,392	73	6.23 × 10^–7^	2.80 × 10^–3^	4
9	5,048,355–5,294,874	36	2.14 × 10^–7^	5.52 × 10^–3^	3
9	15,117,357–15,127,879	48	4.01 × 10^–7^	2.26 × 10^–3^	2
10	3,181,235–3,276,403	55	5.29 × 10^–7^	2.57 × 10^–3^	1
10	20,424,930–20,531,639	105	1.72 × 10^–7^	1.48 × 10^–3^	10
11	543,196–552,444	76	3.91 × 10^–7^	2.24 × 10^–3^	4
11	17,670,561–18,929,198	393	5.41 × 10^–8^	6.79 × 10^–4^	23
12	1,844,142–3,584,210	297	4.13 × 10^–8^	5.99 × 10^–4^	21
13	1,681,154–1,716,546	56	3.46 × 10^–7^	2.13 × 10^–3^	2
13	9,215,174–9,262,342	61	8.65 × 10^–7^	3.29 × 10^–3^	4
14	4,527,908–4,542,487	148	1.03 × 10^–7^	1.01 × 10^–3^	2
14	13,457,733–13,466,285	36	1.14 × 10^–6^	3.83 × 10^–3^	4
16	503,770–637,134	55	2.77 × 10^–7^	1.91 × 10^–3^	5
16	2,405,643–2,750,127	1333	4.31 × 10^–10^	6.35 × 10^–5^	38
17	5,162,885–6,250,543	8	2.16 × 10^–6^	5.56 × 10^–3^	5
18	9,029,424–9,781,214	357	8.04 × 10^–8^	9.20 × 10^–4^	21
19	6,101,292–6,102,285	19	1.50 × 10^–6^	4.52 × 10^–3^	1
20	9,895,217–9,899,561	16	3.11 × 10^–6^	6.83 × 10^–3^	3
20	10,004,382–10,590,764	100	2.78 × 10^–9^	1.64 × 10^–4^	5
22	1,480,237–1,486,672	20	3.37 × 10^–6^	7.14 × 10^–3^	1
22	3,734,510–3,835,387	116	8.65 × 10^–7^	3.29 × 10^–3^	8
22	5,266,531–5,268,855	21	2.89 × 10^–6^	6.56 × 10^–3^	3
23	2,341,704–2,342,263	18	3.01 × 10^–6^	6.71 × 10^–3^	1
23	6,082,331–6,085,084	25	7.15 × 10^–3^	3.00 × 10^–3^	2
25	258,310–296,757	77	1.44 × 10^–7^	1.33 × 10^–3^	6
25	1,537,552–1,537,977	18	1.61 × 10^–6^	4.70 × 10^–3^	1
25	2,210,263–3,017,751	531	3.38 × 10^–8^	5.72 × 10^–4^	24
25	3,699,556–3,707,549	51	5.03 × 10^–7^	2.52 × 10^–3^	3
26	1,166,789–1,182,684	56	5.17 × 10^–7^	2.55 × 10^–3^	1
27	5,189,796–5,371,260	43	2.47 × 10^–6^	6.36 × 10^–3^	2
27	6,481,778–7,699,113	871	2.31 × 10^–11^	8.41 × 10^–6^	36
28	2,456,294–3,326,745	99	4.13 × 10^–7^	2.30 × 10^–3^	8

In total, 305 protein-coding genes, 14 pseudogenes, 12 miRNA, 10 lncRNA, and one snoRNA were detected in putative regions under selection (see Additional file 8: Table S8). Several functional genes were located within the regions that displayed the strongest signals, including genes involved in the immune system (*BF1*, *TAP1*, *DMB2*, *BLB1*, *BLB2*, *TAPBP*, *BRD2*, *BLEC1*, *C4*, *IKZF3*, *DMB1*, and *IL4I1*) and in morphological traits such as the creeper (*IHH* and *NHEJ1*) and comb traits (*MNR2*). In addition, important genes relevant to growth and reproductive traits (*CACNB1*, *STAT5B*, *STAT5A, BCL2L1*, *GNRHR*, *FANCA* and *NTRK1*) were detected in several other significant regions (see Additional file 8 : Table S8). The results of the GO enrichment analyses revealed interesting terms related to basic metabolic activities, such as hyaluronan catabolic process, transport, and hyaluronan biosynthetic process (see Additional file 4: Table S4).

### QTL overlapping with signatures of selection

Quantitative trait loci associated with 179 traits that overlapped with detected signatures of selection were retrieved from the chicken QTL databases (see Additional file 9: Table S9). Analysis of the overlaps between signatures of selection and reported QTL indicated that most of the detected regions contained a QTL (see Additional file 10: Figure S1). Some QTL for traits of economic interest, such as breast muscle weight and percentage, body weight, abdominal fat weight, feed conversion ratio and etc., overlapped significantly with putative regions of signatures of selection (permutation P-value < 0.05). We also found significant overlaps of QTL related to behavioral, morphological, and immune traits (such as feather pecking, drumstick and thigh weight, tibia features, antibody titer to SRBC antigen) with putative regions.

## Discussion

Since the domestication of chickens from the red jungle fowl about 10,000 years ago, evolutionary processes, such as population bottlenecks, migration, inbreeding, and founder effects, have led to a large diversity of chicken populations. A relatively wide range of this diversity is found in the chicken populations of the Iranian plateau and, in this study, we focused on two Iranian native chicken ecotypes, the Lari and Khazak ecotypes. It seems that the Lari ecotype has derived from the Malay breed in the Lar region of the Fars province, Southwest of Iran, in the seventeenth century [35]. According to narrations, the British brought numerous Malay chickens to Iran due to trading between the British and the Iranians on the Persian Gulf shores [35]. The physical similarity of the body shape between the Lari ecotype (long legs and upright posture) and the Malay breed and its dissimilarity with other indigenous ecotypes in Iran support this hypothesis. In contrast, there is no information about the origin of the Khazak ecotype [35, 60].

Through natural selection, local chickens have adapted to harsh environmental conditions, such as heat stress and poor nutrition. In addition, such local chickens are known to represent interesting genetic resources for resistance to local diseases and environments [27, 32]. Muir et al. [61] indicated that pure commercial lines have lost 50% or more of the genetic diversity (especially rare alleles related to resistance to infectious diseases) that existed in their ancestral breeds and consequently, the poultry industry may have to face unforeseen production challenges such as new virulent diseases. Accordingly, native chicken populations are essential for the poultry breeding programs as sources of rare alleles, to maintain the level of genetic diversity in commercial poultry [61]. In addition, these gene resources could be used as an excellent genetic base in genomic selection and crossbreeding programs to develop meat-type and egg-type chickens in local regions. This could be an important source of income for small-holder farmers who raise such indigenous chickens.

### Mapping runs of homozygosity and inbreeding

Analysis of the distribution of ROH and of their length and abundance along the genome can provide valuable information about a population’s history, genomic inbreeding, and signatures of selection [48, 62, 63]. It has been suggested that the higher resolution of whole-genome sequence data than that of SNP chip data could lead to the identification of ROH shorter than 1 Mb [26]. In our study, the relatively low coverage of the whole-genome sequence data may have led to the identification of some incorrect ROH segments. However, Ceballos et al. [48] indicated that SNP chips and low coverage WGS data can achieve equivalent results for ROH calling. Examination of the distribution of ROH in the two indigenous chicken populations showed short to long ROH sizes, ranging from 0.3 to 5.4 Mb. ROH shorter than 1 Mb predominated. Most of the continuous homozygous genotypes were located on macro-chromosomes, while the ROH on microchromosomes were shorter and less numerous, which confirms previous studies in chickens [26, 32, 63]. It should be noted that recombination rates and nucleotide diversity are much higher for the microchromosomes than for the macrochromosomes [64]. In the future, availability of a precise genetic map of the chicken genome based on whole-genome sequence data would allow the distribution of ROH by recombination rates to be appropriately scaled to better interpret the difference in the ROH distribution between the micro- and macrochromosomes.

The average length of the genome covered by ROH per animal (62.32 Mb) was smaller than the genomic ROH coverage reported for a commercial broiler line (130.9 Mb on average) [26], which could be the result of lower inbreeding and a wider gene pool in the Iranian indigenous chicken ecotypes than in commercial lines [32]. The frequency of ROH segments along the genome provides clues about the history and the management of these populations over time [48, 65]. It has been reported that the larger number of ROH in broiler lines could be due to the artificial selection pressure on traits of economic interest [26]. Zhang et al. [66] suggested that differences in *F*_ROH_ values observed between Chinese indigenous, game, and commercial chicken breeds could be due to differences in selection pressure over time. To test this hypothesis, we applied the *F*_ROH_ method to estimate inbreeding within the two populations because it is likely to be the most powerful method to assess inbreeding and it provides more accurate information about the levels of autozygosity than the inbreeding coefficient calculated based on pedigree information [31, 67–69]. When the threshold for ROH size was set to less than 1 Mb, both indigenous ecotypes indicated high levels of inbreeding. This likely reflects ancestral relationships and more ancient inbreeding. The length of the ROH decreases over time due to recombination events, and the presence of short ROH across the genome indicates more distant shared ancestors [49, 70]. The Lari ecotype showed a higher inbreeding levels for all ROH categories and generally a higher level of genome-wide inbreeding than the Khazak ecotype. This may be due to the fact that the local farmers select the Lari chickens mainly for fighting and game purposes, while they raise the Khazak ecotype for egg production, which has not been subjected to any breeding program [34].

Identifying positive signatures of selection can provide valuable information about the influence of selection on adaptive, productive, and morphological characteristics. In this study, we applied four procedures, *F*_ST_ and hapflk (inter-population), and ROH and CLR (intra-population), to identify signatures of selection for two phenotypically different chicken ecotypes. Depending on the nature of the information, each of these statistics has its advantages and disadvantages and may capture a specific pattern of selection [16, 17, 71]. The *F*_ST_ statistic has been widely applied in various studies, is more powerful for the detection of complex events, but it does not take the hierarchical structure of subpopulations into account; this limitation has been fixed in the hapFLK statistic, which is robust with regard to evolutionary processes, such as bottlenecks and migration, and has the ability to uncover a hard selective sweep, i.e. a new beneficial mutation that rises in frequency and spreads quickly to complete fixation. The CLR statistic can detect selective sweeps that are close to fixation, whereas the short ROH (~ 1 Mb) that are detected by consensus ROH (cROH) analysis mostly arose through selective pressure on identical-by-descent genomic regions from distant ancestors [70]. However, short ROH can also result from other evolutionary processes, such as bottlenecks and genetic drift [62, 68]. Therefore, considering ROH regions as signatures of selection should be viewed with caution [72].

### Genes related to growth and reproductive traits

The Lari fighting chickens are characterized by an amazing level of activity. Cockfighting is a form of exercise in which the chicken requires mighty muscle and skeletal structure to run and jump. Thus, we hypothesized that some candidate genes could be connected with muscle growth, hypertrophy, and limb development. Along that line, the two genes *DCN* (*decorin*) and *MEOX2* (*mesenchyme homeobox 2*) were good candidates, as both were detected by three of the four applied methods. *DCN* encodes a connective tissue protein, i.e. a multifunctional proteoglycan that enhances skeletal muscle tissue proliferation and differentiation by interfering with myostatin activity [73]. *DCN* is also involved in modulating collagen assembly and bone mineralization [74]. Previous studies have reported that *DCN* expression increases in response to exercise and muscle contraction, which contribute to muscle hypertrophy [75, 76]. Furthermore, the growth hormone (GH) positively regulates *DCN* in a gender-dependent manner, resulting in greater impact in men than in women [77]. *MEOX2* is a homeobox gene and is associated with skeletal muscle tissue and bone development [78]. In mice, genetic deletion of *MEOX2* reduces muscle mass and causes a developmental defect in the limb musculature [79]. It has been shown that *MEOX2* affects muscle fiber metabolism and muscle size in adult mice [80].

In meat-type chickens, breast muscle is the most valuable carcass component and is one of the most important economic traits. The Lari chickens, with their wide breast and heavy weight (3–4 kg), can be considered as a meat-type chicken. In the haplotype-based analysis (hapFLK), we applied a stringent significant threshold to control false positive signals. One of the most striking signatures of selection was detected in a region that contained the *CACNB1* (*calcium voltage-gated channel auxiliary subunit beta 1*) gene on chromosome 27, with a P-value lower than 2.31 × 10^–11^. *CACNB1* is known to be associated with body weight in broilers [81] and to play an important role in skeletal muscle growth in mice [82].

Compared with meat-type chickens, the low weight of the Khazak chickens could be an asset because their energy consumption for maintenance will be lower and, thus, most of their feed intake will be dedicated to egg production. Gene annotation of the putative regions identified in the cROH and CLR analyses identified the *ESR1* gene on chromosome 3 as a putative signature of selection for reproductive traits in the Khazak ecotype. In birds, the theca cells are responsible for the synthesis of estrogen in the ovarian follicles. Estrogen regulates the synthesis of egg white and yolk proteins, calcium mobilization, and reproductive behavior, and is necessary for folliculogenesis. The effect of estradiol on folliculogenesis is mediated by the estrogen receptors α and β, which are encoded by the *ESR1* and *ESR2* genes, respectively [83]. In the chicken ovary, expression of the *ESR1* gene is higher than that of the *ESR2* gene. *ESR1* is reported to be associated with ovarian functions, especially during follicular development [83, 84], and to be one of the candidate genes for traits related to egg production in chicken and quail [85, 86]. This gene has also been be associated with reproductive traits in pigs and sheep [87, 88].

Both the *F*_ST_ and CLR analyses for the Khazak ecotype revealed another gene that is involved in reproduction traits, *CALCR* (*calcitonin receptor*). The calcitonin receptor binds to calcitonin, a polypeptide hormone, and is associated with regulation of follicular maturation in the chicken ovary [89]. In addition, CALCR is known to play a role in calcium homeostasis during pregnancy in mammals to protect the maternal skeleton [90, 91].

In the putative regions of signatures of selection, several QTL were previously detected for economically important traits, such as growth, abdominal fat weight, drumstick and thigh muscle weight, egg number, and egg production rate. Since no breeding program has ever been implemented to improve production traits in the Iranian indigenous chickens, the identification of genes and QTL that are associated with these traits in these populations may be important for designing breeding plans in the future.

### Genes related to the immune system

Chickens raised in village regions are under different environmental conditions that can affect their productive performance. Natural selection pressure has led to genetic adaptations in village chickens that allow them to survive in the harsh and diverse environmental conditions [25]. It has been demonstrated that ROH regions can harbor candidate genes associated with immune responses and adaptation to severe conditions [31, 63]. We identified several genes and previously detected QTL associated with immune traits in the consensus ROH regions that overlapped with the *F*_ST_ windows. For example, the *S1PR1* (*sphingosine-1-phosphate receptor 1*) gene was identified on chromosome 8 and is known to encode a lipid regulator that is involved in processes such as immune response [92] and to contribute to immune response to viral infections, especially influenza infection [93]. Activation of *S1PR1* can reduce morbidity and mortality in H5N6-infected chickens by suppressing the induction of cytokines, chemokines, and pattern recognition receptors (PRR) [94]. It has also been shown that the *S1PR1* gene plays an essential role in inflammatory responses to infection with the Newcastle disease virus [95, 96]. Another important gene that was detected is *ALPK1* (*alpha-kinase 1*), which is a member of the group of atypical kinase genes [97]. It contributes to innate immune responses to invasive bacteria and influenza virus [98, 99].

In the hapFLK analysis, the strong selective sweep that was detected on chromosome 16 encompasses important genes related to immune traits. Miller and Taylor Jr [100] reported that almost all the genes that are currently mapped to chicken chromosome 16 play a prominent role in immune response. These genes are defined in regional units, of which the best-known region is the major histocompatibility complex-*B* (MHC-*B*). The MHC is a group of polymorphic genes with a central role in the immune system, particularly in resistance to infectious diseases [101–104]. In our study, this haplotype was identified as a putative region under selection and contains the *BF1*, *BF2, TAP1*, *DMB2*, *DMA*, *BLB1*, *BLB2*, *TAPBP*, *BRD2*, *BLEC1*, *C4*, *IKZF3*, *BZFP1*, *CENPA*, *CYP21A1*, *LTB4R*, *HEP21*, *TRIM7.2*, *ZNF692, TRIM7.1*, *TRIM27.1*, *TRIM27.2*, *TRIM39.2*, *TRIM41*, *TRIM39.1*, and *GNB2L1* genes. MHC-*B* haplotypes are important for resistance or susceptibility to infectious diseases such as Marek's disease, Russian sarcoma, and avian leukemia [105, 106].

The population differentiation analyses (*F*_ST_ and hapFLK) revealed potential candidate genes that are relevant to immune traits. This was expected because the Lari and Khazak ecotypes live in different eco-climates. Moreover, because the Lari chickens are used for entertainment and fighting purposes, they have a higher economic value than the Khazak chickens and thus, they probably receive more healthcare from their owners.

### Genes related to morphological and behavioral traits

The Khazak chickens are characterized by a small body size and, compared to the Lari chickens, the length of their leg is much shorter (4 cm vs. 16 cm), such that in the local language, they are known as a creeper chicken. Within the identified putative regions of signatures of selection, two genes that are known to influence the creeper trait were detected in the whole-genome scan using the haplotype differentiation analysis. The strongest signal was observed on chromosome 7 between 22.29 and 22.32 Mb, which included 79 SNPs. This region spans two important genes, *IHH* (*Indian hedgehog*) and *NHEJ1* (*non-homologous end-joining factor 1*), and has been documented to be the causal genes of the creeper trait in chickens [107, 108]. The creeper trait is determined by a single autosomal gene in chickens that results in shortened legs [109]. Recently, Kinoshita et al. [107] demonstrated that a 25-kb deletion that harbors the *IHH* and *NHEJ1* genes on chromosome 7 has a significant role in the *Cp* phenotype of Japanese chickens.

Aggressive behavior has been evolutionarily conserved during chicken domestication [110] and is preserved and reinforced in the Lari chicken, which have been bred and trained to fight. Three approaches (*F*_ST_, CLR, and ROH) identified the same signature of selection on chromosome 2, which has the highest m*F*_ST_ value (0.541) and a significant CLR value (8.80). This consensus ROH region was present in 73.3% of the Lari population analyzed. This region includes the *AGMO* (*alkylglycerol monooxygenase*) gene, which encodes the only enzyme that cleaves the *O*-alkyl bond of ether lipids and plays a role in the disabilities and neurodevelopmental disorders in humans [111, 112]. Luo et al. [113] highlighted the presence of strong selective sweeps for the *AGMO* gene that are associated with behavioral patterns in the Chinese gamecock chickens. We also detected the *GNAO1* gene in a cROH region on chromosome 11 that was present in 47% of the Lari chickens. It has been shown that the *GNAO1* gene is involved in the aggressive behavior of the Xishuangbanna fighting chicken and is under selection in that population [114, 115]. Another identified candidate gene related to behavioral traits was *PSEN1* (*presenilin 1*), which is located on chromosome 5 and was detected in the *F*_ST_ and CLR analyses of the Khazak ecotype. The *PSEN1* gene has a role in neuropsychiatric diseases in humans [116] and may contribute to feather pecking behavior [117].

Since each statistical approach is able to detect specific signatures of selection across the genome, it is possible that they do not detect the same region for a given signature of selection [27]. However, Almeida et al. [26] reported that about half of the detected *F*_ST_ windows overlapped with the detected cROH across the genome. In our study, we detected several putative regions of signatures of selection and genes that were shared by at least two of the four methods used, but we did not observe overlapping regions between the *F*_ST_ and hapFLK approaches because these two methods recognize specific different patterns of signatures of selection. The detection of shared regions under selection by different methods could provide persuasive evidence about the effect of selection on a specific region in the genome, which may be associated with critical functional traits. In addition, the putative regions of signatures of selection that overlap with known QTL can enhance the accuracy of detected signatures of selection [28]. However, to validate these regions, more evidence is required, such as from scanning replicated populations, functional analysis, and positional cloning.

## Conclusions

We implemented four intra and inter-population methods to detect signatures of selection using whole-genome resequencing data in two indigenous chicken ecotypes. Our results revealed several putative footprints of selection that harbor candidate genes associated with disease resistance, growth, reproductive, morphological, and behavioral traits. Although indigenous chicken ecotypes are not always considered for large-scale commercial purposes, they have good potential to survive in harsh environmental conditions. Our findings enhance our understanding of the relationships between phenotype differentiation and genotypes among different breeds.

## Supplementary Information

**Additional file 1: Table S1.** Summary of the distribution of ROH lengths along the genome per animal. An excel file containing the number of ROH, total size of genome covered by ROH (kb) and average of ROH length (kb) per animal.

**Additional file 2: Table S2.** List of candidate genes that overlap with regions identified by the CLR method in the Lari and Khazak ecotypes.

**Additional file 3: Table S3.** List of the genes that were identified simultaneously by at least two of the four applied methods. This table represents the identified common genes shared by at least two of the four methods used, *F*_ST_, hapFLK, CLR and ROH, (L: Lari ecotype and K: Khazak ecotype).

**Additional file 4: Table S4.** Summary of GO terms related to biological processes resulting from the analyses of cROH regions, CLR, *F*_ST_ and hapFLK in the Lari and Khazak ecotypes. This table represents gene ontology (GO) terms that are significant (P-value > 0.05), (L: Lari ecotype and K: Khazak ecotype).

**Additional file 5: Table S5.** List of consensus ROH pools for each ecotype. This file contains information about family identification (FID), individual identification (IID), chromosome (CHR), start position (BP1) and end position (BP2), segment size (KB) and number of SNPs in each ROH.

**Additional file 6: Table S6.** List of candidate genes that overlap with regions identified by the ROH method in the Lari and Khazak ecotypes.

**Additional file 7: Table S7.** List of candidate genes that overlap with regions identified by the *F*_ST_ method.

**Additional file 8: Table S8.** List of candidate genes that overlap with regions identified by the hapFLK method.

**Additional file 9: Table S9.** Overlaps between the reported QTL in the QTL database and the detected candidate regions by *F*_ST_, hapFLK, CLR and ROH analysis. This file presents the overlaps between the traits and the position of the known QTL regions in chicken with the position of the identified putative signatures of selection by the four methods.

**Additional file 10: Figure S1.** Results of the overlap analysis between the regions of signatures of selection (set A) and known QTL regions in chicken (set B). The y-axis shows the number of overlaps between the two datasets. AinB refers to the region in A encompassed by a region in B; BinA refers to the region in B encompassed by a region in A; AleftB and ArightB refer to the end and the start of the region of signatures of selection that overlap with the beginning and the end of a region of QTL, respectively.

## Data Availability

The datasets used for the current study are available from the corresponding author upon reasonable request.
