# Correction: A genome-wide scan to identify signatures of selection in two Iranian indigenous chicken ecotypes

**DOI:** 10.1186/s12711-022-00720-y

**Published:** 2022-04-19

**Authors:** Elaheh Rostamzadeh Mahdabi, Ali Esmailizadeh, Ahmad Ayatollahi Mehrgardi, Masood Asadi Fozi

**Affiliations:** Department of Animal Science, Faculty of Agriculture, Shahid Bahonar University of Kerman, 22 Bahman Blvd, Kerman, Iran

## Correction to: Genet Sel Evol (2021) 53:72 https://doi.org/10.1186/s12711-021-00664-9

After publication of this work [1], we noticed that (1) we had not clearly stated that the data are publicly available and (2) we had not cited the original paper [2]. Therefore, the following sections “Background”, “Methods”, “Availability of data and materials” and “References” are incomplete and should be amended as follows.

## Background

Page 1, first paragraph of the “Background” section where we stated “that domestic chicken originated from the Red Jungle fowl (*Gallus gallus*) in the south and Southeast Asia”, the following sentence is missing:

“Recently, a comprehensive study, which analyzed 863 whole-genome sequences from a worldwide sampling of chickens and representatives of all four species of wild jungle fowl and each of the five subspecies of red jungle fowl (RJF), concluded that domestic chickens were initially derived from the RJF subspecies *Gallus gallus spadiceus* whose present-day distribution is predominantly in southwestern China, northern Thailand and Myanmar [2]”.

## Methods

### Sampling and genome sequencing

Page ‘3’, first paragraph, line ‘10’ where we described the preparation of DNA samples, the following sentence is missing:

“Whole-genome sequencing of the samples using the next-generation sequencing technique was conducted within the framework of the Global Chicken Genome Project (http://chicken.ynau.edu.cn/index/about/index.html) led by the Kunming Institute of Zoology, Chinese Academy of Sciences (KIZ-CAS) and described by Wang et al. [2–5]”.

### Availability of data and materials

The following sentence “The datasets used for the current study are available from the corresponding author upon reasonable request” should read:

“The datasets used for the current study were from Wang et al. [2]. The raw sequencing data, alignment of BAM files, and genotypes (VCF) are available in the ChickenSD database (http://bigd.big.ac.cn/chickensd/)”.
