# Supplementary material for: A genome-wide scan to identify signatures of selection in two Iranian indigenous chicken ecotypes
Source: Genet Sel Evol. 2021 Sep 9;53:72. doi: 10.1186/s12711-021-00664-9 (PMC8428137; doi:10.1186/s12711-021-00664-9)
Supplement: Supplementary file 10 — Additional file 10: Figure S1. Results of the overlap analysis between the regions of signatures of selection (set A) and known QTL regions in chicken (set B). The y-axis shows the number of overlaps between the two datasets. AinB refers to the region in A encompassed by a region in B; BinA refers to the region in B encompassed by a region in A; AleftB and ArightB refer to the end and the start of the region of signatures of selection that overlap with the beginning and the end of a region of QTL, respectively. [file 12711_2021_664_MOESM10_ESM.docx]

Fig. 1 The result of overlap analysis between the selection signature regions (set A) and known QTLs in chicken (set B). The y-axis shows the number of overlaps between two data set. AinB refers to the region in A encompassed by a region in B; BinA refers to the region in B encompassed by a region in A; AleftB and ArightB refer to the end and the start of the region from signature selection regions overlapped with the beginning and the end of a region in QTL regions, respectively.
